# Supplementary material for: Effects of Inflammatory Factor Expression Regulated by 12/15 Lipoxygenase on Obesity-Related Nephropathy
Source: Nutrients. 2022 Jun 30;14(13):2743. doi: 10.3390/nu14132743 (PMC9268756; doi:10.3390/nu14132743)
Supplement: Supplementary file 1 [file nutrients-14-02743-s001.zip › nutrients-1691653-supplementary.pdf]

Figure S1

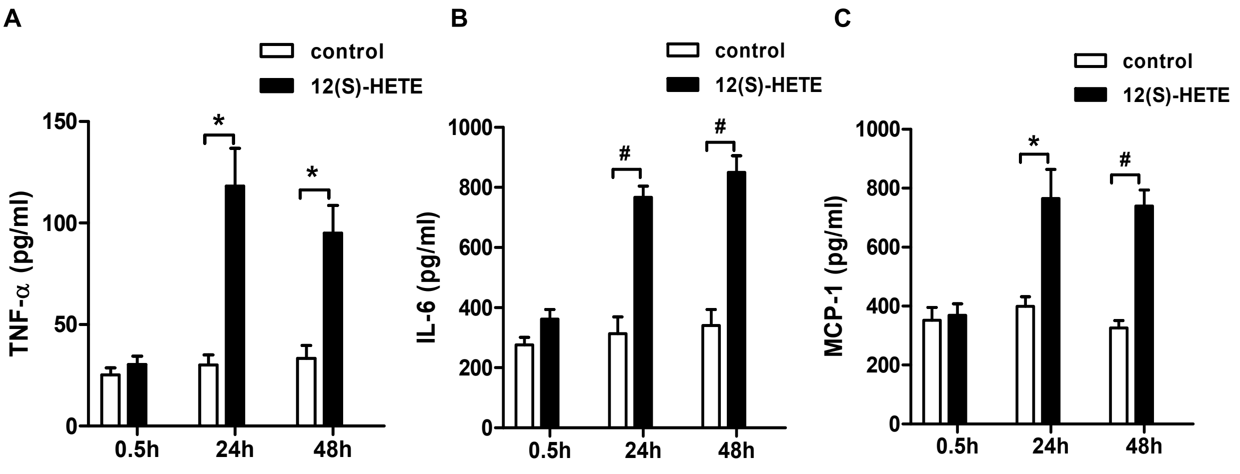

Figure S1. Effects of 12(S)-HETE on the production of adipose inflammatory factors. Equal numbers of subconfluent MCs were cultured in medium (RPMI1640+1%FBS) containing either buffer (control) or 12(S)-HETE (10^{-7}M) for 0.5-48 h. An aliquot supernatant was collected from each well and content of TNF-α (A), IL-6 (B), and MCP-1 (C) were measured by ELISA kits. Results are from three sets of experiments and shown as fold over control (mean±SEM, n=3. *p<0.05, #p<0.01 vs. control).

Figure S2

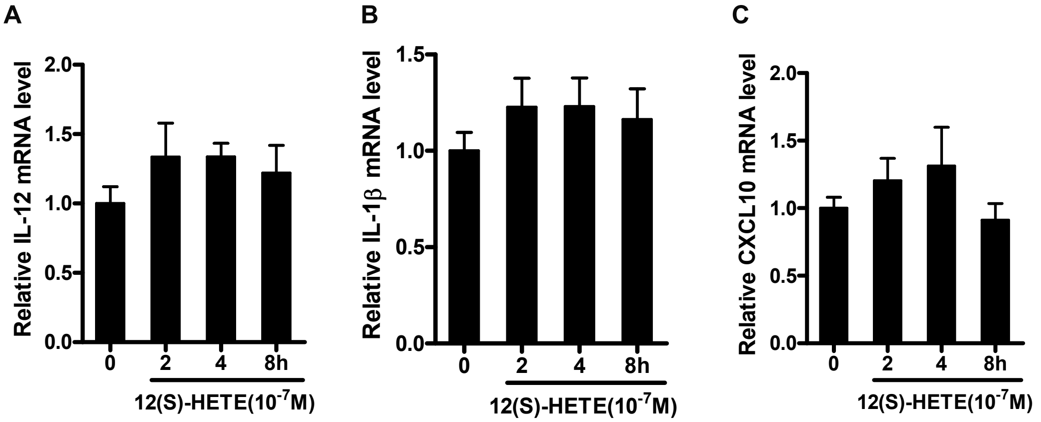

Figure S2. Effects of 12(S)-HETE on the expression of adipose inflammatory factors. MCs were stimulated with 12(S)-HETE (10^{-7}M) for 2-8 h and changes in intracellular mRNA levels of IL-12 (A), IL-1β (B), and CXCL-10 (C) were measured by RT-qPCR. Results are expressed as fold over none treatment of control (mean±SEM, n=3).

Figure S3

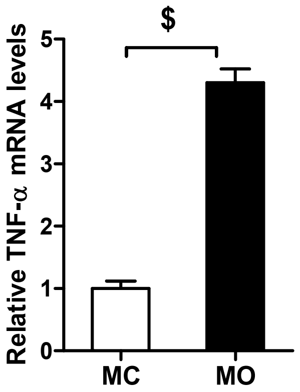

Figure S3. Quiescent MCs and MOs were collected for experiment. Intracellular mRNA expression of TNF-α was measured by RT-qPCR, β-Actin was used as internal control. Results are expressed as fold over MCs (mean±SEM, n=3. $p<0.001 vs. MC).

Figure S4

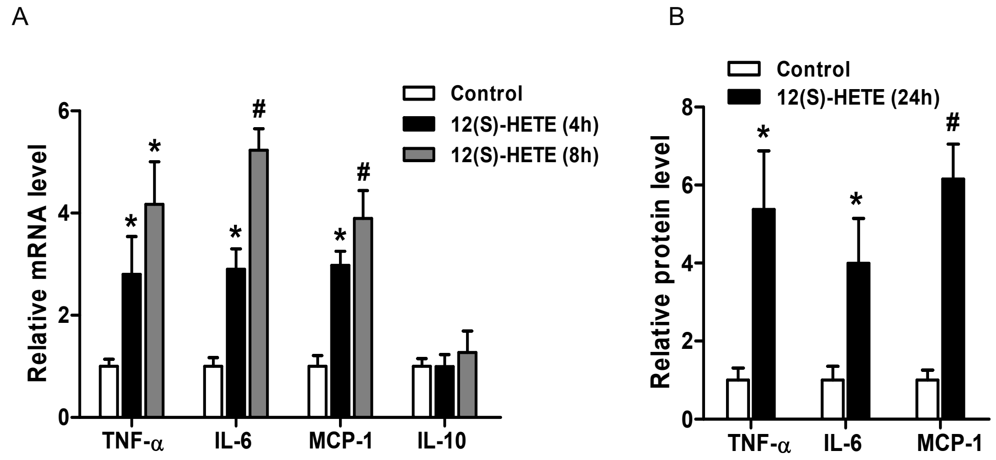

Figure S4. Effects of 12(S)-HETE on the expression of adipose inflammatory factors in MOs. (A) Quiescent MOs (RAW264.7) were stimulated with 12(S)-HETE (10^{-7}M) for 4-8 h and changes in intracellular mRNA levels of TNF-α, IL-6, MCP-1 and IL-10 were measured by RT-qPCR. Results are expressed as fold over none treatment of Control (mean±SEM, n=3). (B) Equal numbers of subconfluent MOs were cultured in medium (DMEM+1%FBS) containing either buffer (Control) or 12(S)-HETE (10^{-7}M) for 24 h. An aliquot supernatant was collected from each well and content of TNF-α, IL-6, and MCP-1 were measured by ELISA kits. Results are from three sets of experiments and shown as fold over Control (mean±SEM, n=3. *p<0.05, #p<0.01 vs. Control).

Table S1. Primer sequences used in RT-qPCR analysis

	Primers (5'→3')	
**Gene (species)**	**Sense**	**Anti-sense**
IL-1β (M)	TTGACGGACC CCAAAAG ATG	AGAAGGTGCTCATG TCCTCA
IL-12 (M)	TCTGCAGAGAAGGTCACACT	ATGAAGAAGCTGGTGCTGTA
CXCL10 (M)	CTCATCCTGCTGGGTCTGAG	CCTATGGCCCTCATTCTCAC

M, Mouse
